# Evolution of Modeled Cortisol Is Prognostic of Death in Hospitalized Patients With COVID-19 Syndrome

**DOI:** 10.3389/fmed.2022.912678

**Published:** 2022-06-06

**Authors:** Kamyar M. Hedayat, David Chalvet, Maël Yang, Shahrokh Golshan, Caroline Allix-Beguec, Serge Beneteaud, Thomas Schmit

**Affiliations:** ^1^Systems Biology Research Group, Chicago, IL, United States; ^2^Numa Health International, La Rochelle, France; ^3^Department of Psychiatry, University of California, San Diego, San Diego, CA, United States; ^4^Centre Hospitalier de La Rochelle, La Rochelle, France; ^5^Emergency Medicine, Centre Hospitalier de La Rochelle, La Rochelle, France

**Keywords:** COVID-19, models, biological, cortisol index, neutrophil/lymphocyte ratio (NLR), C-reactive protein, critical care outcomes

## Abstract

**Introduction:**

Patients hospitalized with SARS-CoV-2 have an elevated risk of mortality related to a severe inflammatory response. We hypothesized that biological modeling with a complete blood count (CBC) would be predictive of mortality.

**Method:**

In 2020, 81 patients were randomly selected from La Rochelle Hospital, France for a simple blinded retrospective study. Demographic, vital signs, CBC and CRP were obtained on admission, at days 2-3 and 3-5. From a CBC, two biological modeling indexes were resulted: the neutrophil-to-lymphocyte ratio (NLR) and cortisol index adjusted (CA).

**Results:**

By ANOVA, in survivors vs. non-survivors there was statistical different at *p* < 0.01 for age (66.2 vs. 80), CRP (92 vs. 179 mg/dL, normal < 10), cortisol index adjusted (323 vs. 698, normal 3-7) and genito-thyroid indexes (7.5 vs. 18.2, normal 1.5–2.5), and at *p* = 0.02 creatinine (1.03 vs. 1.48, normal 0.73–1.8 mg/dL). By mixed model analysis, CA and NLR improved in those who survived across all three time points, but worsened again after 3–5 days in non-survivors. CRP continued to improve over time in survivors and non-survivors. Positive vs. Negative predictive value were: CRP (91.1%, 30.4%), NLR (94.5%, 22.7%), CA (100%, 0%).

**Discussion:**

Cortisol modeling and the neutrophil-to-lymphocyte ratio were more accurate in describing the course of non-survivors than CRP.

**Conclusion:**

In patients admitted for SARS CoV-2 infection, biological modeling with a CBC predicted risk of death better than CRP. This approach is inexpensive and easily repeated.

## Introduction

The COVID-19 pandemic has an estimated 0.6% mortality rate per total infections in the US, and 0.4% in France, both prior to the omicron variant (1, 2). However, due to the high incidence (66% estimated for the US population), the absolute number of hospitalized patients and the burden on tertiary levels of care is substantial. Furthermore, once hospitalized, mortality is approximately 20% (3). Many patients who die in-hospital initially improve before a final, fatal deterioration. Identifying these patients prior to their fatal deterioration may reduce intensive care admission and possibly mortality.

Inflammation is the common denominator in the mechanisms resulting in death, be it acute respiratory distress syndrome (ARDS), sepsis or end-organ failure (4). Many studies have already associated inflammatory burden with mortality. It remains unclear which markers of inflammation are most strongly correlated and at what point of hospitalization. C-Reactive Protein (CRP) is a well-studied marker of acute inflammatory reactant in patients with COVID-19 (5). However, there are other indicators or actors in acute inflammation. For example, the neutrophil-to-lymphocyte ratio (NLR) (6–10) is associated with mortality on admission for patients with COVID-19 (7). It is has been referred to as the genito-thyroid index (GTi) in an attempt to describe the upstream endocrine mediators of neutrophil and lymphocyte production (6). Another marker is serum cortisol. High serum cortisol at admission to the hospital was associated with higher mortality in one study (11), but the opposite has also been observed (12). Tissue levels have not consistently been associated with severity of illness (13, 14). Our group (KMH, JCL) has demonstrated that the effective tissue-level activity of cortisol can be modeled using a complete blood count with differential (CBC) (15) in chronic heart failure and acute myocardial infarction (AMI) (16, 17) and that the model of admission cortisol activity was superior to serum cortisol in predicting mortality in AMI (17). The formation of the index is based on the observation that cortisol increases erythrocytes, leukocytes, neutrophils, and monocytes, and, it diminish lymphocytes, eosinophils, and the eosinophil/monocyte ratio (18–21). On this basis, the elements of a CBC that are increased are in the numerator and those diminished are in the denominator of the formula that composes the basic cortisol index.

At the time of study design, we were not aware of a study that compared these three approaches to inflammation to each other. We sought to evaluate changes in biomarkers associated with inflammation to determine (1) at what point in the first 5 days of hospitalization would factors associated with deterioration or death be present, and (2) to determine which biomarker or indexes related to inflammation best predicted this deterioration or death.

## Method

**FIGURE 1:**
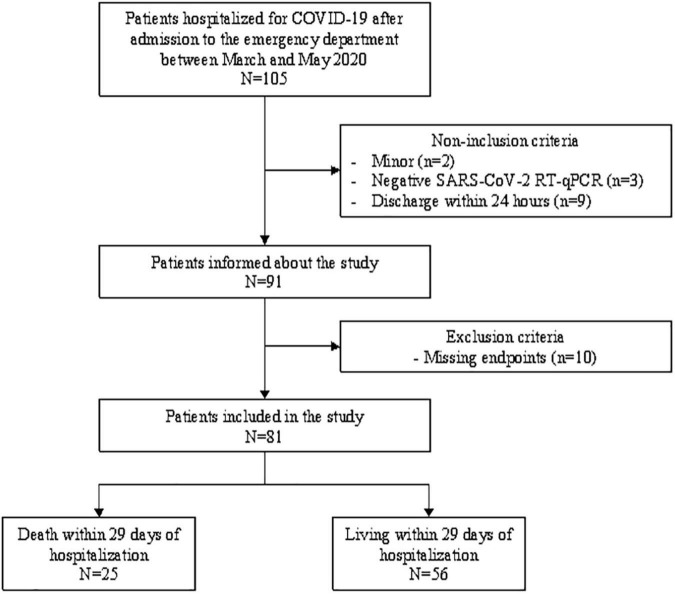
Patient selection and longitudinal monitoring process.

From March through May, 2020, 91 patients admitted to an emergency department in South-West France were recruited for a simple blinded retrospective study (Figure 1), of which 81 agreed to participate. The inclusion criteria were: positive SARS CoV-2 diagnosis by PCR and age > 17. The exclusion criteria were admission less than 24 h, pregnancy at the time of admission, and, surgery, chemotherapy or glucocorticoids within the prior 2 weeks before admission. After approval from the hospital’s ethics committee and patients’ information, coded, anonymized data was entered regarding age, sex, blood pressure, oxygen saturation, survival status, CRP, and a CBC with differential from the time of admission, days 2-3 and 3-5. Survival or death were determined by criteria summarized in Figure 1.

Numa Health International prepared the anonymized data for Systems Biology Research Group (SBRG). SBRG used Microsoft Excel (v16.16.25, Microsoft, Seattle, Washington, DC, United States) to calculate the GTi (NLR) and cortisol index. Because of the non-parametric distribution of values of the cortisol index in this study, SBRG developed a proprietary formula that conditionally corrects individual biomarkers used to derive the formula. The formula for the cortisol index is discussed elsewhere (22) and summarized in the introduction. The corrected formula modified these elements using bijection once their value was above or below a certain extreme value. Statistical analysis was performed using SPSS V24 (SPSS, Chicago, IL, United States). The Kolmogorov–Smirnov test was used to determine normal distribution of data prior to analysis.

## Results

All 81 hospitalized patients were positive for SARS CoV-2. The mean age was 69.4. There were 56 men and 31 women. The all-group mortality rate was 28.7% (25/87). Men died at nearly twice the rate of women 35.7% vs. 16.1% (*p* < 0.01). The following factors were found to be statistically non-significant: sex, smoking history, hypertension, renal disease, diabetes mellitus type 2, or number of medications regularly consumed at time of admission. By analysis of variance (ANOVA), the admissions values of creatinine, CRP, cortisol index adjusted and genito-thyroid indexes (NLR) were all statistically significant between survivors and those who died (Table 1). Admission status or values of the following were not found to be statistically significant: hemoglobin (*n* = 82), platelets (*n* = 82), body mass index (*n* = 44), sex (*n* = 82), smoking status (*n* = 17), poly medication (*n* = 59), hypertension (*n* = 35), diabetes mellitus type 2 (*n* = 22), chronic respiratory illness (*n* = 19).

**TABLE 1:** ANOVA of admission variables.

Variable (normal value)	Total number	Survivors (mean value)	Died (mean value)
Age* (years)	81	66.2	80
Creatinine** (0.73–1.18 mg/dL)	81	1.03	1.48
CRP* (<10 mg/dL)	78	92	179
Cortisol adjusted* (3–7)	76	323	698
Genito-thyroid* (1.5–2.5)	76	7.5	18.2

*p < 0.01, **p = 0.02.

Logistic regression of admission values for CRP, cortisol index adjusted and the genito-thyroid index (NLR) showed very good positive predictive value but poor negative predictive value (Table 2).

**TABLE 2:** Logistic regression of indicators of inflammation.

Variable	PPV	NPV
CRP*	51/56 (91.1%)	7/16 (30.4%)
GTi (NLR)**	52/56 (94.5%)	5/17 (22.7%)
Cortisol index adjusted*	55/55 (100%)	0/21 (0%)

*p = 0.002, **p = 0.005.

*GTi, Genito-thyroid index; NLR, neutrophil-lymphocyte ratio; NPV, negative predictive value; PPV, positive predictive value.*

**FIGURE 2:**
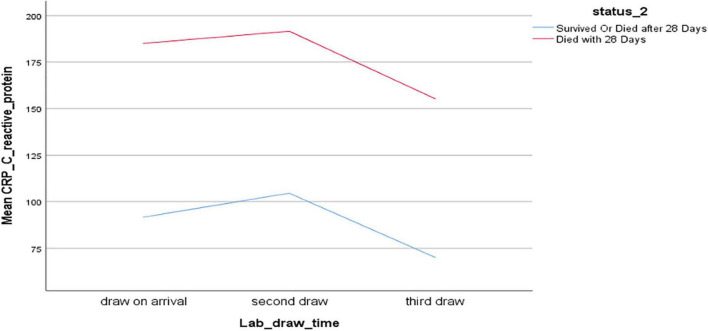
Mixed model analysis of C-reactive protein (mg/dL), time of blood draw, and survival.

Next, we used a mixed model analysis to evaluate any significant trends across the first (admission), second (day 2-3) and third blood draws (day 3-5). CRP was higher in those who died than in the survivors, but in both groups it worsened between first and second draw, and improved between second and third draw (Figure 2).

**FIGURE 3:**
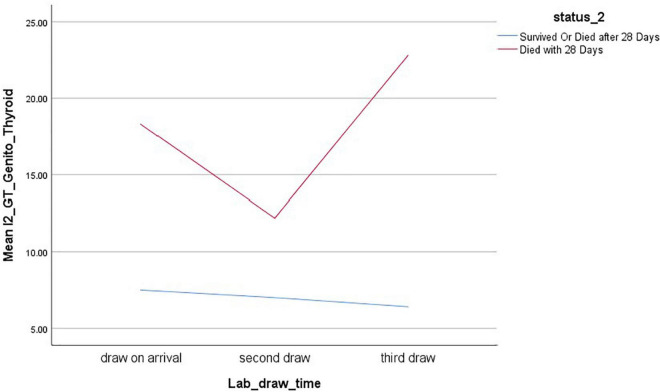
Mixed model analysis of genito-thyroid index (Neutrophil-to-Lymphocyte ratio, no units), time of blood draw, and survival.

**FIGURE 4:**
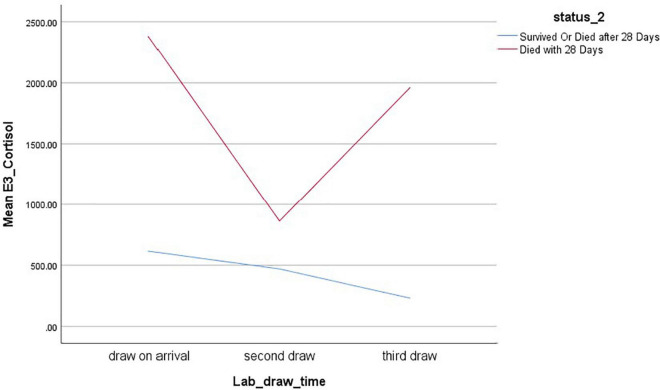
Mixed model analysis of Cortisol index adjusted (no units), time of blood draw, and survival.

In contrast, the genito-thyroid and cortisol adjusted indexes showed continued improvement in those who survived, but a sudden worsening after improving in those who died, with the mean final value being worse than that at admission (Figures 3, 4).

## Discussion

The three markers of inflammation we studied represent different facets of inflammation. CRP and NLR represent humoral immunity-mediated inflammation. The neutrophil to lymphocyte ratio (NLR), also referred to as the genito-thyroid index (GTi) is a more generalized expression of inflammation, being a ratio of two types of leukocytes (10, 15, 23). CPR is a more specific but indirect reflection of humoral-immunity-induced inflammation. Produced in the liver, monocytes and T-lymphocytes excrete interleukin-6, which stimulates CRP release. Finally, cortisol is an adrenal cortex hormone that stimulates tissue-level inflammation through genomic and non-genomic actions (24), such as increased activity of NF-κB. Thus, it represents a key endocrine mediator of inflammation and more largely adaptation.

All three inflammatory markers differed significantly between survivors and non-survivors at admission. However, the evolution of CRP did not differ between those who died and those who survived. It progressively improved in both groups. In our study, GTi (NLR) differed significantly on admission for those who survived and died, and worsened only in the group who died. Others have found that the admission value of GTi (NLR) was also associated with worse outcomes (7, 8, 25). The cortisol index adjusted showed results similar to GTi (NRL), but represents a new approach to modeling inflammation in patients with COVID-19. The latter two are rapid, cost effective, and easily repeated throughout hospitalization. We (KMH, JCL) have demonstrated the value of this approach to modeling in predicting mortality on admission for patients admitted with acute myocardial infarction (17).

None of these three variables had good negative predictive value, which makes their use limited in predicting who is likely to survive. However, admission scores of all three showed strong association with death and the two modeling indexes showed an evolution consistent with sudden worsening prior to death. A future study should use multi-variate analysis to evaluate the interaction between renal failure (creatinine) with these three inflammatory markers in a larger, multi-center trial to determine if a combination of one or more markers of inflammation and kidney function together provides a better negative predictive value on admission.

There are short-comings associated with this study that limit applicability of its findings to the current phase of the COVID-19 pandemic. Our study was a small single-center retrospective study. We had insufficient data to stratify risk of mortality by number of co-morbidities or severity of illness. Because the hypothesis was to evaluate the physiologic terrain and the use of biological modeling, we did not obtain information about clinical presentation which may have yielded additional insights into risk factors for death. The study conducted prior to the onset of more robust variants of SARS CoV-2 and prior to the availability of a vaccine. Finally, while the unadjusted cortisol index has been studied in other disorders (20, 22), the adjusted version has not.

## Conclusion

In patients with SARS CoV-2, on admission, CRP, neutrophil-to-lymphocyte ratio (genito-thyroid index) and the cortisol adjusted index were significantly different between those who survived and died. The latter two showed improvements then worsening in those who died, while CRP did not. In this small, single-center retrospective study, indexes modeling systemic inflammation and derived from the commonly performed CBC with differential showed better association with evolution of illness than CRP in patients admitted with COVID-19.

## Data Availability Statement

The original contributions presented in the study are included in the article/supplementary material, further inquiries can be directed to the corresponding author.

## Ethics Statement

The studies involving human participants were reviewed and approved by the Emergency Department of Centre Hospitalier de La Rochelle. The patients/participants provided their written informed consent to participate in this study.

## Author Contributions

KMH: principle investigator, study conception, interpretation of data, and manuscript preparation. DC: study coordinator, interpretation of data, and manuscript preparation. MY: data analysis, data visualization, and manuscript preparation. SG: statistical analysis and interpretation of data. CA-B: study conception, study site coordinator, and data acquisition. SB: study conception and study administration. TS: manuscript preparation. All authors contributed to the article and approved the submitted version.

## Conflict of Interest

KMH is a shareholder in SBRG and Numa Health. DC, TS, and MY are shareholders in Numa Health. KMH serves as a paid advisor to Numa Health. The remaining authors declare that the research was conducted in the absence of any commercial or financial relationships that could be construed as a potential conflict of interest.

## Publisher’s Note

All claims expressed in this article are solely those of the authors and do not necessarily represent those of their affiliated organizations, or those of the publisher, the editors and the reviewers. Any product that may be evaluated in this article, or claim that may be made by its manufacturer, is not guaranteed or endorsed by the publisher.
